# Dual-targeting and steric hindrance resolution in HER2 IHC: a novel approach to improve diagnostic sensitivity

**DOI:** 10.1186/s12885-025-14553-7

**Published:** 2025-07-29

**Authors:** Li Luo, Xi Zhang, Linqiong Chen, Zhuohan Chen, Yuchen Wang, Kaihao Huang, Xiaoyun Lin, Hongxiang Zhu, Wangqi Du

**Affiliations:** 1 Department of Clinical Laboratory, Taixing People’s Hospital Affiliated to Yangzhou University, Taizhou, China; 2 School of Basic Medical Sciences, Wenzhou Medical University, Cha Mountain Street of High Education Park, Ouhai District, Wenzhou, 325000 China; 3 Department of Chemotherapy and Radiotherapy, The Second Affiliated Hospital and Yuying Children’s Hospital of Wenzhou Medical University, Wenzhou, China; 4 The Alberta Institute, Wenzhou Medical University (Joint Institute), Wenzhou, China; 5 Department of Pathology, Taixing People’s Hospital Affiliated to Yangzhou University, Taizhou, China

**Keywords:** HER2, Breast cancer, IHC, Steric hindrance, Diagnostic sensitivity

## Abstract

**Background:**

The HER2 immunohistochemistry (IHC) test is an essential method for detecting breast cancer (BC) and plays a pivotal role in guiding personalized treatment strategies. However, inconsistencies persist among different pathologists using IHC, especially for HER2-low and HER2-negative. This may lead to discrepant clinical decisions, potentially impacting patient outcomes. Since HER2 exists in both dimeric and monomeric forms in cells, certain binding sites of diagnostic antibodies on HER2 dimers may be partially obscured in detection. Therefore, accurately detecting HER2 dimers in IHC is crucial for improving diagnostic precision.

**Methods:**

We aligned the structures of HER2 heterodimers and Fabs of pertuzumab and trastuzumab binding to HER2, and found they binding in the same region. To overcome the steric hindrance of HER2 dimers, we employed HER2-binding affibody (Aby) and nanobody (Nby) to construct their fusion protein (Nby-Aby) and human heavy chain ferritin (HFn) based nanoparticles (Nby-HFn, Aby-HFn) for detection. Since the Nby and Aby bind HER2 at two distinct regions that are separate from the HER2 dimerization region, effectively minimizing interference from HER2 dimerization in detection. We assessed the detection performance of Nby-Aby in BC tissues and compared it with conventional HER2 diagnostic antibodies using tissue microarrays (TMAs).

**Results:**

The Nby-Aby assay had higher detection sensitivity for HER2-positive cells in BC tissues compared to the conventional method. Additionally, significantly higher HER2 scores were observed in most BC tissues on tissue microarrays (TMAs) compared to those diagnosed using the traditional method. These findings suggest that dual-targeting and overcoming steric hindrance in HER2 IHC detection is a promising strategy to enhance diagnostic precision.

**Conclusions:**

Dual-targeting different regions and overcoming steric hindrance of HER2 in IHC detection through the Nby-Aby fusion protein enhances diagnostic sensitivity, providing a novel strategy for more accurate HER2 IHC assessment in BC diagnosis.

**Supplementary Information:**

The online version contains supplementary material available at 10.1186/s12885-025-14553-7.

## Introduction

The human epidermal growth factor receptor (EGFR) family, also known as the HER or ERBB family, belongs to the category of tyrosine kinase receptors. It consists of four members: EGFR (also known as HER1), HER2, HER3, and HER4 [1]. Dysregulation of these proteins can facilitate aberrant cell proliferation, invasive behavior, and uncontrolled cellular viability, ultimately culminating in carcinogenesis [2]. Among these receptors, HER2 is unique in that it lacks known ligands and mainly heterodimerization with other HER family proteins or homodimerization with other HER2 molecules in a ligand-independent manner for activation [3]. After dimerization, HER2 also triggers the activation of downstream signaling pathways, thereby inducing sustained cellular proliferation and ultimately leading to carcinogenesis [4]. Thus, theoretically, HER2 exists in two states within cells: the unactivated resting monomers and the activated working dimers [5], and the working HER2 dimers in cancer cells are theoretically more than that in normal cells, especially in HER2-related cancer cells.

Breast cancer (BC) is the second most prevalent malignancy among women, and HER2 overexpression in BC is often associated with an aggressive phenotype and poor clinical outcomes [6]. IHC and in situ hybridization (ISH) are commonly employed for the evaluation of HER2 overexpression levels in BC, with IHC being widely acknowledged as a cost-effective, time-efficient, and user-friendly method, particularly in resource-limited settings where ISH confirmation may be constrained [7]. Numerous studies consistently demonstrated a high level of concordance between IHC and ISH, with an average agreement rate exceeding 90% [8, 9]. Therefore, IHC is still an effective and more convenient method compared to ISH for the assessment of HER2 expression levels in BC.

Previously, according to the guidelines set by the American Society of Clinical Oncology (ASCO) and the College of American Pathologists (CAP), HER2-positive was defined as an IHC score of 3 + or a score of 2 + with ISH +, while HER2-negative included HER2 zero, HER2 1 +, and HER2 2 + with ISH- [10]. The HER2-positive patients were considered eligible candidates for interventions such as trastuzumab, whereas the HER2-negative patients were deemed unsuitable [11]. This emphasizes the importance of accurate diagnosis of HER2-positive or HER2-negative for further intervention, particularly in cases when patients are diagnosed with equivocal HER2 IHC 2 +. In such cases, an ISH test should be employed as a complementary assessment [12], so the precise distinction of HER2 scores 2 + using IHC is crucial.

Interestingly, recent clinical trials have demonstrated that trastuzumab deruxtecan (T-DXd) is effective in patients with HER2-low metastatic BC [13], with modest anti-tumor activity also observed in HER2 ultra-low or even zero patients [14]. This highlights the significance of accurately distinguishing between HER2 IHC scores of zero and low [15], as detecting HER2 IHC scores of 0 or 1 + is meaningful for identifying patients eligible for T-DXd treatment, despite lacking formal endorsement [16]. However, the concordance rate of HER2 IHC scores of 0 and 1 + in the same tissue among different pathologists may be low, with only 26% consensus among 18 pathologists. In contrast, agreement substantially increases to 70% for scores of 2 + and 3 + [17]. Moreover, even in the completed surveys, about 48% of the HER2 IHC 0 slides were found to have detectable HER2 expression [14], and 67% of cases classified as HER2 IHC 0 showed detectable HER2 expression through quantitative immunofluorescence [18]. Collectively, these findings highlight the importance of precise HER2 diagnosis using IHC, particularly for scores of 0 and 1 +, as it serves as a crucial strategy to reduce patients'misclassification for T-DXd or other treatments [17].

The HER2 molecule consists of an extracellular domain (ECD), a transmembrane domain (TMD), and an intracellular domain (ICD), and the ECD is further subdivided into four subdomains: ECD I, ECD II, ECD III, and ECD IV [19]. Similar to other HER members, HER2 dimerization relies mainly on the exposed ECD II [20], and thus the dimerization of HER2 may present challenges in accurately evaluating HER2 levels through IHC. It was reported that the three most well-known diagnostic antibodies used in IHC tests may primarily target the same region of HER2 [21], and their binding regions may be obscured when HER2 molecules are in the dimerization conditions once they bind in the same region. In such conditions, developed antibodies used for tests may not bind with HER2 dimers but only monomers due to the steric hindrance effects, potentially leading to an inaccurate assessment of HER2. Thus in IHC tests, the antibody used for binding of regions different from HER2 dimerization to avoid steric hindrance is crucial.

Given this issue, to overcome HER2 dimer steric hindrance and enhance detection accuracy in IHC, we employed two HER2 binding molecules, namely affibody (Aby, Z_HER2:342_) [22] and nanobody (Nby, 2Rs15d) [23] to construct the fusion protein Nby-Aby. Since both the Nby and Aby possess unique binding sites different from the regions required for HER2 dimerization and those of certain antibodies targeting HER2 [24, 25], Nby-Aby may dual-targeting HER2 and meanwhile not be affected by HER2 dimers in IHC detection. Also, Nby [26] is currently undergoing phase II clinical trial evaluation (NCT03924466), and Aby has demonstrated potential effects on HER2-positive tumors both in vitro and in vivo [27, 28]. Therefore, the development of Nby-Aby for IHC testing may have the potential to enhance the accuracy of detection. Meanwhile, to further enhance their diagnostic sensitivity and for easier production in future applications, the human heavy chain ferritin (HFn) nanoparticles of Nby and Aby were also constructed, and each nanoparticle may consist of 24 Nby or Aby monomers after self-assembly [29].

The results of the present study demonstrated that the Nby-Aby assay, compared with the conventional method, exhibited the capability to identify tumor cells that were undetected by the antibody in IHC diagnosis. Meanwhile, in comparison to the initially diagnosed data on TMAs, the Nby-Aby assay also demonstrated a significant increase in HER2 scores in the tissues initially diagnosed as HER2 zero and 1 +, but consistently aligned with the antibodies diagnosed when the HER2 score exceeded 2 +. Therefore, by dual-targeting and binding with different regions to overcome the steric hindrance of HER2 dimers in IHC tests, our research introduces a novel method and direction that may offer increased sensitivity in detecting HER2.

## Methods

### Materials

The construction, sequencing identification, and transformation of the plasmids were facilitated by Sangon Biotech Co., Ltd. (Shanghai, China). Ni–NTA agarose resin was purchased from Qiagen (Dusseldorf, Germany). Isopropyl-D-thiogalactopyranoside (IPTG) and ampicillin powder were obtained from Generay (Shanghai, China). Prestained protein ladder was sourced from Thermo Fisher (Massachusetts, USA). Phosphate buffer solution (PBS), healthy goat serum, Tween-20, and Bicinchoninic acid (BCA) kit were provided by Beyotime (Beijing, China). Diaminobenzidine (DAB), HRP-conjugated goat anti-rabbit IgG antibody, endogenous peroxidase blocking solution (3% hydrogen peroxide), and hematoxylin were all procured from ZSGB-Bio (Beijing, China). HER2 IHC assay rabbit primary antibody was acquired from Roche Diagnostics GmbH (Tucson, USA), and Rabbit anti-HA monoclonal antibody was obtained from Proteintech (Wuhan, China). The BC tissues and TMAs were from the Outdo Biotech Co., Ltd (Shanghai, China), and all the tissues were sourced from the Shanghai National Engineering Research Center for Biochips in China, along with the initial diagnostic information (HER2 scores, ISH, Ki67, tumor sizes) supplied.

### Structural alignment analysis of HER2-binding proteins

To verify our hypothesis that HER2 dimers may have steric hindrance in certain cases, the crystal structures of the HER2 extracellular domain (ECD, PDB: 1n8y) and its interactions with EGFR (PDB: 8hgo), HER3 (PDB: 7mn5), HER4 (PDB: 8u4k), pertuzumab and trastuzumab Fab (PBD: 6oge), Nby (PDB:5my6), and Aby (PDB:3mzw) were individually retrieved from the Protein Data Bank database (https://www.rcsb.org/). Subsequently, ligands and water molecules were removed, followed by aligning all other structures with HER2-ECD. The aligned structures were then visually depicted in distinct colors to facilitate analysis of their respective binding regions.

### Construction of dual-targeting Nby-Aby and HFn-based nanoparticle plasmids

To construct the recombinant plasmids for fusion protein production, the gene sequences of Nby (PDB: 5MY6_B), Aby (GenBank: AEN27854.1), and HFn (NP_002023.2) were searched on PubMed, followed by optimization of prokaryotic expression for further research. To construct the dimer, the gene sequences of Nby and Aby were connected with a glycine (4)-serine (G4S) linker in the middle, and a hemagglutinin tag (HA tag) was added at the N-terminal for detection. To construct the nanoparticles, genes encoding Nby and Aby were separately linked to the N-terminal of HFn using a glycine (4)-serine (G4S) linker for connection, along with an additional HA tag for detection. The recombinant genes, in conjunction with HFn genes, were then chemically synthesized by Sangon Biotech Co., Ltd. (Shanghai, China). Subsequently, they were individually inserted into pET21a(+) plasmids using *Nde*I and *Xho*I as restriction sites to construct the recombinant pET21a(+)/HFn, pET21a(+)/Nby-Aby, pET21a(+)/Nby-HFn, and pET21a(+)/Aby-HFn plasmids.

### Expression and identification of Nby-Aby molecules and HFn-based nanoparticles

To prepare the fusion proteins for IHC testing use, the pET21a(+)/HFn, pET21a(+)/Nby-Aby, pET21a(+)/Nby-HFn, and pET21a(+)/Aby-HFn plasmids were individually transformed into *E.coli* BL21(DE3) after successful sequencing verification. Following induction with 1 mM IPTG at 37℃ for 6 h, bacterial sediments were collected and dissolved in PBS for ultrasonic lysis. The resulting supernatants were purified using Ni–NTA agarose chromatography and the eluted proteins were dialyzed in PBS after being eluted with 50 mM imidazole. Subsequently, protein concentrations were determined using the bicinchoninic acid (BCA) method while samples from the dialysis solution underwent SDS-PAGE analysis. Further, transmission electron microscopy (TEM) was performed to examine the prepared nanoparticle samples. In brief, purified Nby-HFn, Aby-HFn, and HFn proteins (0.1 mg/mL) were applied to carbon-coated copper grids and incubated for 2 min. The grids were rinsed with ultrapure water, stained with 2% (w/v) uranyl acetate for 1 min, and air-dried. TEM imaging was performed using a Hitachi HT-7800 microscope at 110 kV and × 100,000 magnification. A 100 nm scale bar was included during image acquisition. The remaining protein solutions were aliquoted and stored at −80 °C for future use.

### The selection of candidate reagents for the IHC test

To select the optimal fusion protein for IHC and verify our hypothesis that binding different regions in HER2-ECD can avoid steric hindrance of HER2 dimers and enhance sensitivity, a three-step IHC procedure was conducted. The formalin-fixed and paraffin-preserved pathological tissue samples of BC were provided by the Outdo Biotech Co., Ltd and rediagnosed at Taixing People's Hospital (Jiangsu, China). Unstained sections with a thickness of 4 μm were cut and subsequently baked at 65 °C for 1 h. Following deparaffinization, antigen retrieval, and peroxidase blocking, antigens were blocked by healthy goat serum (Solarbio, Beijing, China) for 30 min. After being washed with PBST three times, the sections were incubated separately overnight at 4℃ with 200 μg/mL of HFn, Nby-Aby, Nby-HFn, and Aby-HFn. Meanwhile, PBS was utilized as a negative control, and the conventional anti-HER2 antibody (Con-Ab) was used as a positive control at a working concentration of 6 μg/mL. Afterward, all the tissues were washed with PBST. The detection of protein binding was conducted using a rabbit anti-HA tag mAb at a dilution of 1:200, incubated at 37℃ for 2 h as the primary antibody, and followed by the HRP-conjugated goat anti-rabbit IgG as the secondary antibody. The bound peroxidase was visualized using DAB and the nucleus was stained with hematoxylin. Finally, the sections were dehydrated and sealed for subsequent detection.

### Nby-Aby molecules were used for TMA analysis

To validate the enhanced sensitivity in detecting HER2 within BC specimens, Nby-Aby molecules were carefully selected and conjugated with HRP for subsequent IHC analysis on tissue microarrays (TMAs). Firstly, the Nby-Aby molecules were conjugated with activated HRP reagents (InnoReagents, Huzhou, China) following the instructions of manufacture. Briefly, a total of 1 mg purified Nby-Aby was dissolved in 2 mL PBS along with 200 μL of starter solution and incubated at 37℃ for 2 h in a dark place. Then, 200 μL of stop solution was added and incubated at room temperature for 1 h to stop the reaction. The resulting mixture was dialyzed in PBS for 2 h.

Secondly, after dewaxing, antigen retrieval, and internal peroxidase blocking, the antigens on TMAs were further blocked with healthy goat serum for 1 h and washed with PBST. Subsequently, Nby-Aby was added and incubated with TMAs at 37℃ for 2 h followed by washing with PBST. Finally, the TMAs were stained separately with DAB and hematoxylin, dehydrated, and sealed for detection. HER2 scores were reassessed by a pathologist at Taixing People's Hospital and the data were collected.

### Stitacal analysis

The HER2 IHC scores reassessed by Nby-Aby were compared with the initial HER2 diagnostic data, and the correlation between them with indicators of ISH, Ki67, and tumor sizes was calculated. The different groups were analyzed using Fisher’s Exact Test in SPSS 22.0 (IBM, America), and *P* < 0.05 was considered statistically significant.

### Prediction of the structures of Nby-HFn, Aby-HFn, and Nby-Aby

To explore the underlying reasons for the unexpected outcomes of nanoparticles in the three-step IHC method, we used the alphafold to predict the structures of Nby-Aby, Nby-HFn, and Aby-HFn. The predicted PBD files were then downloaded, together with the HFn crystal structures (PBD:5n27) from the PBD database. Subsequently, the predicted structures with the highest scores from Nby-HFn and Aby-HFn files were duplicated as 24 separate files, individually aligned with the 24 monomers of HFn, and visualized collectively alongside Nby-Aby using PyMOL.

## Results

### Nby and Aby binding HER2-ECD different from HER family proteins, pertuzumab and trastuzumab Fabs

**Fig. 1:**
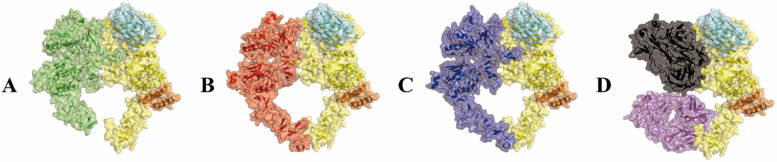
Nby and Aby have distinct binding domains on HER2-ECD compared to other HER family proteins, pertuzumab, and trastuzumab Fab fragments. **A** Structural alignment of EGFR (green), Nby (cyan), and Aby (orange) in complex with HER2-ECD (yellow). **B** Structural alignment of HER3 (red), Nby (cyan), and Aby (orange) in complex with HER2-ECD (yellow). **C** Structural alignment of HER4 (blue), Nby (cyan), and Aby (orange) in complex with HER2-ECD (yellow). **D** Structural alignment of pertuzumab (black) and trastuzumab (magenta) Fab fragments, along with Nby (cyan) and Aby (orange), bound to HER2-ECD (yellow)

After downloading all available crystal structures from the PBD database and aligning the binding regions of Nby and Aby with EGFR (Fig. 1A), HER3 (Fig. 1B), HER4 (Fig. 1C), pertuzumab and trastuzumab Fab (Fig. 1D), we observed a spatial separation between the binding regions of Nby and Aby with HER2-ECD compared to all of them. Nby and Aby predominantly bind to domains I and III within HER2-ECD, which are distinct from both the dimerization region of HER family proteins (domain II) and the binding sites of some Fabs (domain II) on HER2.

### Expression and identification of Nby-Aby and the HFn-based nanoparticles

**Fig. 2:**
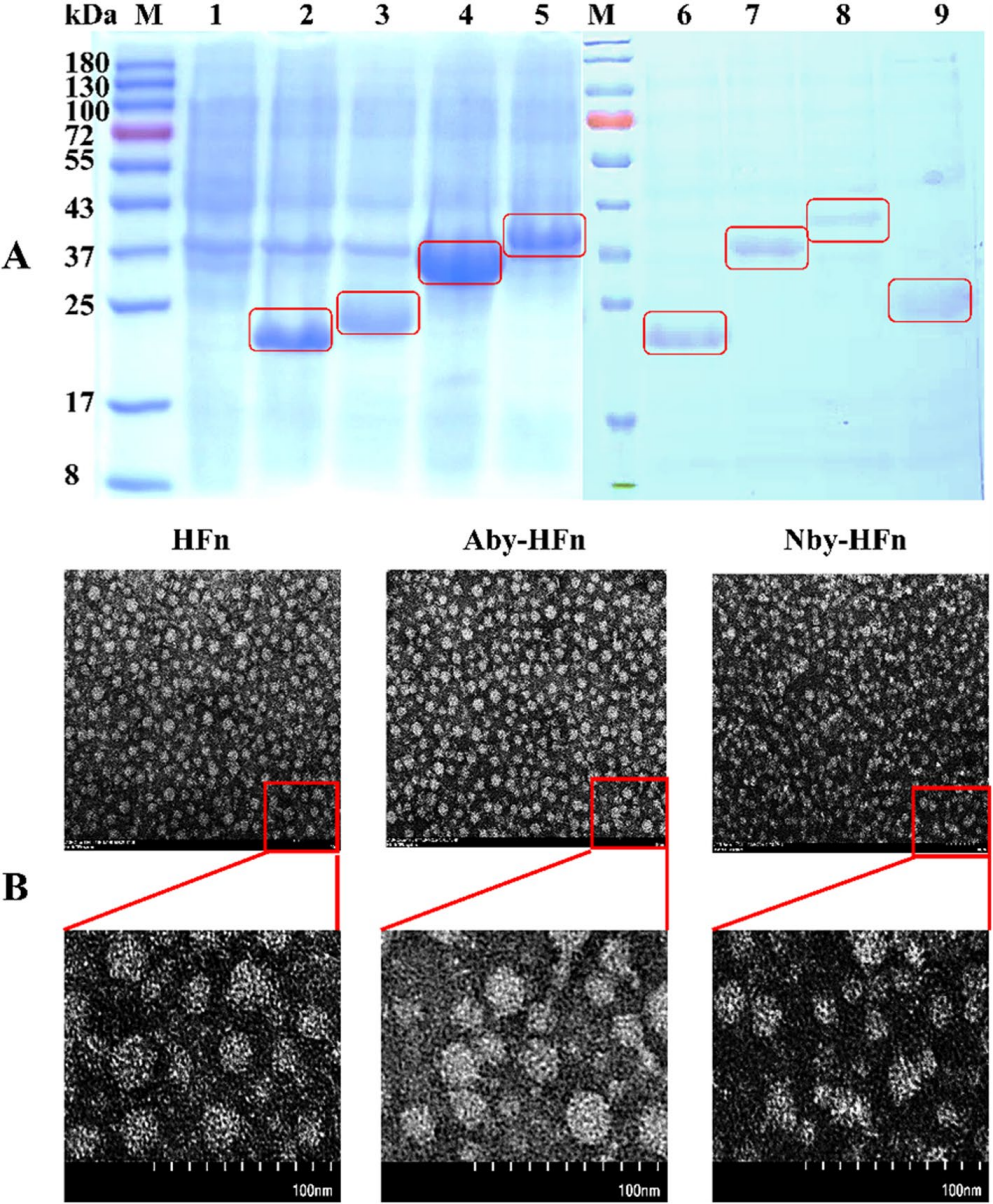
Preparation and characterization of Nby-Aby fusion proteins and HFn-based nanoparticles. **A** SDS-PAGE analysis of induced protein expression in *E. coli* BL21(DE3) and purified recombinant proteins. M: Prestained protein ladder; lane 1: *E. coli* BL21(DE3) without induction; lane 2: Nby-Aby expressed in *E. coli* BL21(DE3); lane 3: HFn expressed in *E. coli* BL21(DE3); lane 4: Aby-HFn expressed in *E. coli* BL21(DE3); lane 5: Nby-HFn expressed in *E. coli* BL21(DE3); lane 6: purified Nby-Aby; lane 7: purified Aby-HFn; lane 8: purified Nby-HFn; lane 9: purified HFn. **B** Transmission electron microscopy (TEM) analysis confirming the correct self-assembly of HFn-based nanoparticles, including HFn, Aby-HFn, and Nby-HFn. scale bars = 100 nm

After plasmids construction (Supplementary Fig. 1) and transfected into *E.coli* BL21(DE3), when induced with 1 mM IPTG and identified by SDS-PAGE, the induced *E.coli* BL21(DE3) showed bands (Fig. 2A) at the molecular weights of 23 kDa (Nby-Aby), 25 kDa (HFn), 34 kDa (Aby-HFn), 40 kDa (Nby-HFn) separately. While the uninduced *E.coli* BL21(DE3) had no bands at the corresponding position. Also, the purified proteins exhibit distinct bands at their respective positions (Fig. 2A). The TEM identified that the HFn, Aby-HFn, and Nby-HFn all formed nanoparticles (Fig. 2B).

### Nby-Aby exhibited higher sensitivity in the HER2 IHC test than the conventional antibody and the HFn-based nanoparticles

**Fig. 3:**
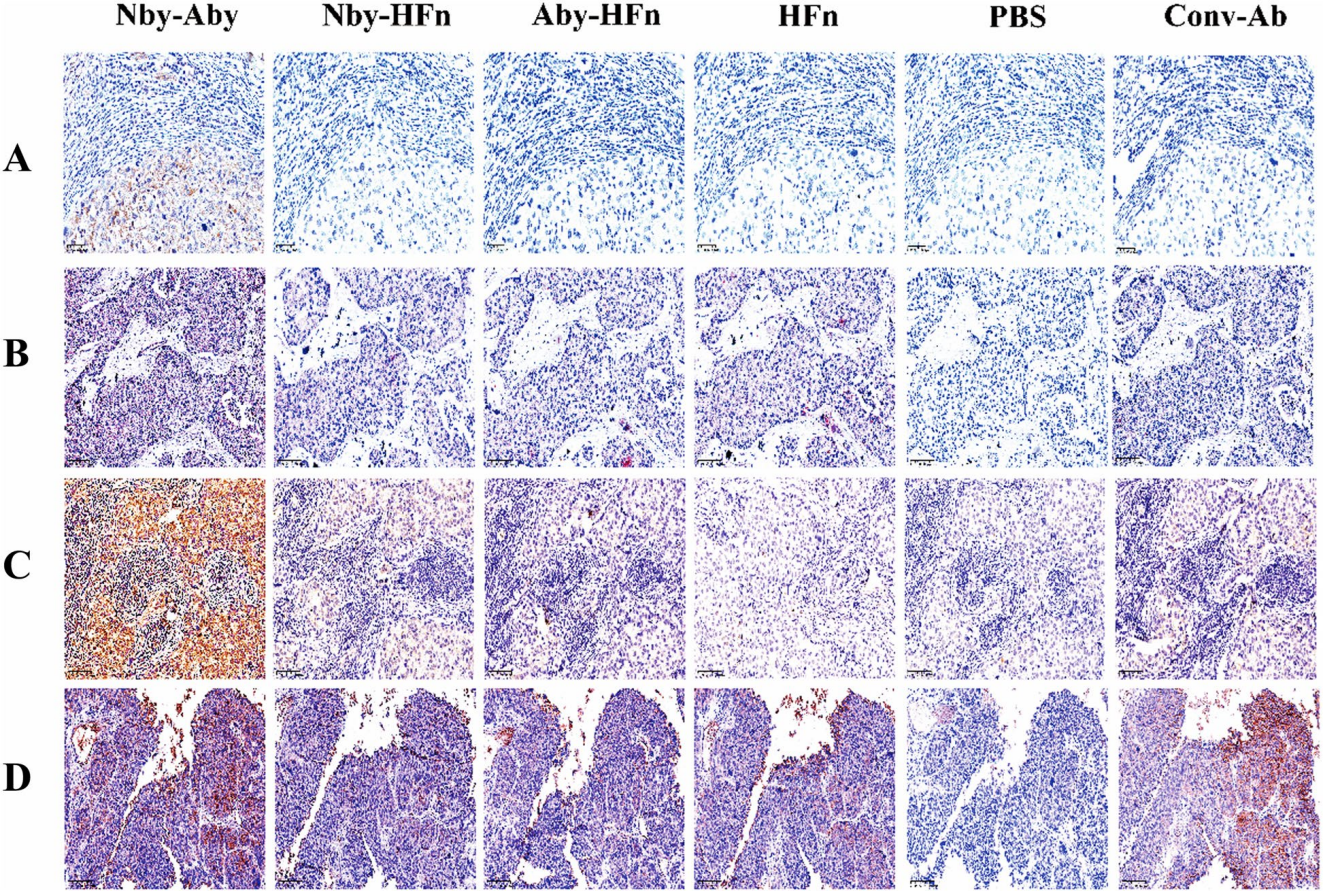
Comparative immunohistochemical analysis of HER2 expression using diverse protein probes and conventional antibody (Conv-Ab). IHC staining images of breast cancer tissue samples originally classified as (**A**) HER2 0, (**B**) HER2 1 +, (**C**) HER2 2 +, and (**D**) HER2 3 + according to standard IHC diagnosis with Conv-Ab. Each row shows comparative staining results obtained using Nby-Aby, Nby-HFn, Aby-HFn, HFn alone, PBS (negative control), and Conv-Ab (positive control). The Nby-Aby probe demonstrates enhanced detection capability compared to Conv-Ab, HFn and PBS groups showed negligible background. Scale bars = 100 μm

We utilized Nby-Aby, Aby-HFn, Nby-HFn, HFn, and PBS for IHC testing and compared the results with the conventional assay. The results showed that in case 1 (Fig. 3A), the HER2 score was determined as zero by the conventional assay, but reevaluated as 2 + by the Nby-Aby test, and Nby-Aby also identified cancer cells that were not detected by the conventional assay. In case 2 (Fig. 3B), the conventional assay was identified as 1 + but was reassessed as 2 + by Nby-Aby. In case 3 (Fig. 3C), scores 2 + were made by the conventional assay but were rescored as 3 + by the Nby-Aby assay. In case four (Fig. 3D), all tests of proteins yielded nearly the same results as the conventional assay with scores of 2 + or 3 +. So, the results validated our hypothesis that using Nby-Aby binding in different regions to overcome steric hindrance in HER2 IHC for detection can enhance the scores of HER2 through IHC.

### Nby-Aby enhanced the HER2 positive rate in IHC compared to the conventional antibodies

**Fig. 4:**
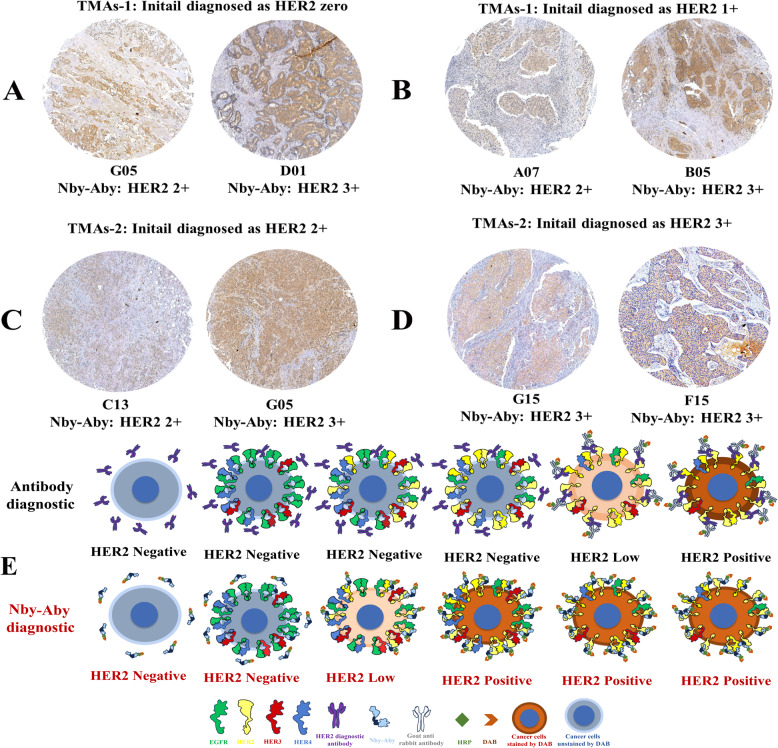
Reassessment of HER2 IHC scores using Nby-Aby and proposed mechanism for scores enhancement. **A** Tissues initially diagnosed by con-Ab as HER2 0 were reassessed as HER2 2 + or 3 + using Nby-Aby. **B** Tissues initially diagnosed by con-Ab as HER2 1 + were reassessed as HER2 2 + or 3 + using Nby-Aby. **C** Tissues initially diagnosed by con-Ab as HER2 2 + were reassessed as HER2 2 + or 3 + using Nby-Aby. **D** Tissues initially diagnosed by con-Ab as HER2 3 + were reassessed as HER2 3 + using Nby-Aby. **E** Schematic illustration of the potential mechanism by which Nby-Aby enhances HER2 IHC scores compared to the conventional antibody

The Nby-Aby molecules were subsequently conjugated with HRP to minimize potential interference from other antibodies and evaluate the sensitivity elevation of the HER2 IHC test. After incubation with Nby-Aby and DAB staining, we observed that in TMAs-1 samples all initially diagnosed as 0 and 1 +, Nby-Aby accurately identified cancer cells within the tissues, and upgrading HER2 IHC scores from 0 (Fig. 4A) or 1 + (Fig. 4B) to 2 + and 3 +. While in TMAs-2 tissues initially diagnosed as HER2 2 +, Nby-Aby also confirmed the diagnoses or upgraded the results to 3 + (Fig. 4C). Similarly, tissues initially diagnosed as HER2 3 + were still 3 + (Fig. 4D). The potential reason that Nby-Aby enhanced the HER2 IHC scores and resulted in enhanced positive rate were shown (Fig. 4E).

The total IHC HER2 scores of Nby-Aby diagnosed and initially diagnosed data were collected and compared, combined with the supplementary data (ISH, Ki67%, and tumor sizes). When combining HER2 scores with ISH, the IHC positive and low (HER2 1 +, 2 +, 3 +) rates of the initial diagnosed group were 64%, while the Nby-Aby diagnosed was 89%, and indicated a highly significant difference (*P* < 0.001). When combining HER2 scores with Ki67 or tumor sizes, the results showed a significant difference (*P* < 0.05) in the groups with Ki67 > 30% and tumor sizes ≤ 2 cm for the Nby-Aby diagnosis compared to the conventional method. This suggests that Nby-Aby may more accurately detect tumors with potentially more aggressive characteristics, demonstrating that the Nby-Aby used for HER2 IHC tests can enhance the sensitivity than the traditional method. The detailed information is shown in Table 1.

**Table 1:** Comparisions between HER2 expression levels assessed by Nby-Aby diagnostics and initial diagnostics combined with other tumor indicators

Characteristics	Initial diagnostic	Nby-Aby diagnostic			Total (%)	Fisher’s exact test	*P-*value
		**Positive (%)**	**Low (%)**	**Negative (%)**			
HER2 expression levels with ISH	Positive (%)	9 (9.0)	0 (0.0)	0 (0.0)	9 (9.0)	12.682	0.009
	Low (%)	26 (26.0)	25 (25.0)	4 (4.0)	55 (55.0)		
	Negative (%)	14 (14.0)	15 (15.0)	7 (7.0)	36 (36.0)		
	Total (%)	49 (49.0)	40 (40.0)	11 (11.0)	100 (100.0)		
HER2 expression levels with Ki67 > 30%	Positive (%)	7 (11.67)	0 (0.00)	0 (0.00)	7 (11.67)	9.898	0.028
	Low (%)	12 (20.00)	13 (21.67)	1 (1.67)	26 (43.33)		
	Negative (%)	11 (18.33)	11 (18.33)	5 (8.33)	27 (45.0)		
	Total (%)	30 (50.00)	24 (40.0)	6 (10.0)	60 (100.0)		
HER2 expression levels with tumor sizes ≤ 2 cm	Positive (%)	4 (10.00)	0 (0.00)	0 (0.00)	4 (10.00)	8.287	0.049
	Low (%)	10 (25.00)	10 (25.00)	3 (7.5)	23 (57.50)		
	Negative (%)	2 (5.00)	8 (20.00)	3 (7.5)	13 (32.50)		
	Total (%)	16 (40.00)	18 (45.0)	6 (15.0)	40 (100.0)		

### Predicted structures of Nby-HFn, Aby-HFn, and Nby-Aby

**Fig. 5:**
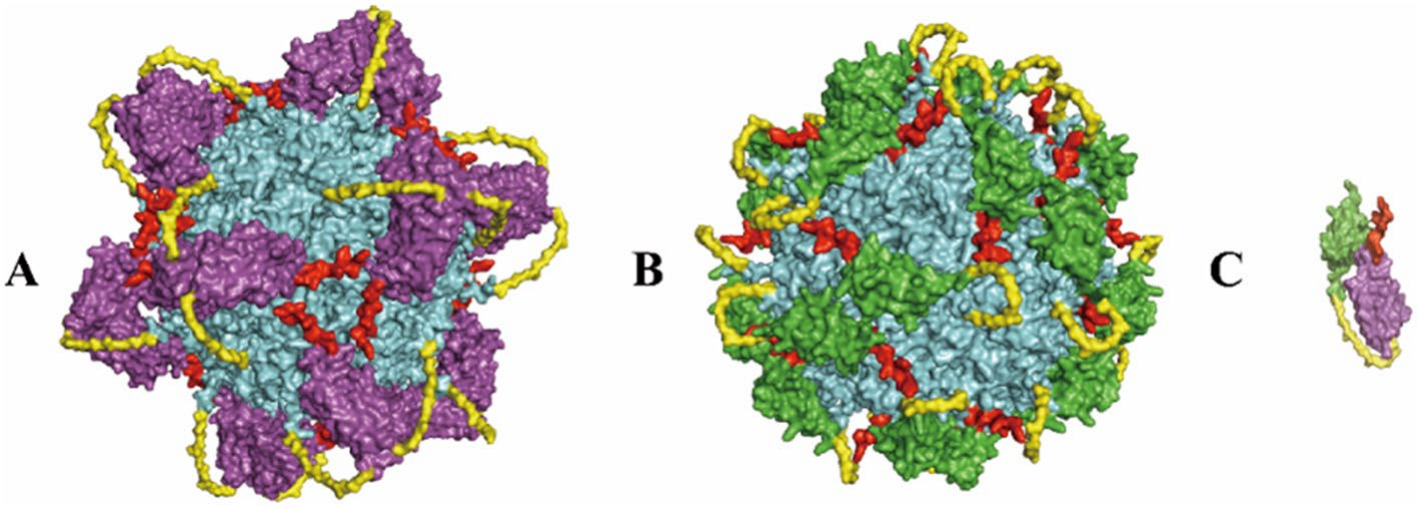
Predicted structures of Nby-HFn, Aby-HFn, and Nby-Aby. **A** Predicted structure of Nby-HFn. **B** Predicted structure of Aby-HFn. **C** Predicted structure of Nby-Aby. Amino acids are color-coded as follows: Nby in magenta, Aby in green, HFn in cyan, G4S-linker in yellow, and the HA tags in red

To investigate the potential reasons behind the inconsistent results of nanoparticles compared to our expectations, we employed the alphafold to predict the crystal structures of Nby-HFn, Aby-HFn, and Nby-Aby. Our analysis revealed that although both Nby-HFn and Aby-HFn successfully self-assembled into nanoparticles as intended, their HA tags were inadequately exposed for binding with HA tag antibodies (Fig. 5A, B). This limited accessibility hinders the effective binding of the HA antibody during the three-step assay despite each nanoparticle containing 24 detectable HA tags. In contrast, the size of the Nby-Aby molecule was significantly smaller than that of nanoparticles (Fig. 5C), and the HA tag remained unshielded. This may explain the discrepancy between our expectations and the results of HER2 binding nanoparticles in the three-step method, despite their design aiming to theoretically enhance sensitivity.

## Discussion

The discovery of HER2 in 1985 has sparked extensive research, resulting in substantial advancements [30]. In 1998, a significant milestone was achieved with the FDA's approval of Trastuzumab for targeted chemotherapy in BC patients. Since then, over 2.3 million individuals have received this treatment, resulting in enduring benefits for many [31]. However, although various therapies have been developed for targeting HER2, the majority of these treatments are exclusively applicable to patients with HER2-positive tumors [32], as for HER2-negative patients, treatment options are limited. Despite this, HER2-positive BC is still considered to be highly invasive and prone to recurrence and metastasis, with limited availability of effective predictive models for assessing the risk of recurrence [33]. Therefore, developing novel methods for more accurate assessment of recurrence risk in BC patients is crucial.

One plausible explanation for the unfavorable prognosis of HER2-positive patients is the role of HER2-mediated signaling in promoting migration and invasion of BC cells [34]. Even among patients undergoing triplet therapy (dual HER2-targeted with chemotherapy), the pathologic complete response was only 45.8% [35], and still unsatisfactory. This may be attributed to the fact that, while pertuzumab and trastuzumab bind distinct ECD of HER2 and effectively inhibit dimerization, they do not completely prevent all forms of HER2 dimerization [36], particularly those pre-existing in cancer cells. Consequently, activated HER2 dimers that persist during treatment may contribute to therapeutic resistance and poor prognosis.

So, we believe that activated HER2 dimers, which are not inhibited by pertuzumab and trastuzumab during the progression of treatment, may still play a role in resistance and poor prognosis. Therefore, accurate detection of HER2 dimerization represents a promising strategy for predicting patient outcomes. Interestingly, Gregory Weitsman and colleagues introduced a FLIM-FRET technique for quantifying HER2-HER3 dimers in BC tissues, demonstrating that the dimerization of HER2 and HER3 is associated with metastatic relapse in BC patients [37]. Similarly, Melanie Spears et al. employed Proximity Ligation Assays (PLAs) to evaluate the presence of HER2-HER2 and HER2-HER3 dimers in BC tissues and demonstrated that both HER2 gene-amplified and non-amplified BC tissues exhibited detectable levels of HER2 dimers, and the elevated levels of HER2 dimers are associated with larger tumor sizes [38]. These findings suggest that evaluating HER2 dimerization could improve prognostic assessments and guide treatment decisions. Therefore, evaluating the levels of HER2 dimers in BC tissues may be useful in predicting recurrence and metastasis in patients in the future.

In this study, we employed Nby-Aby for IHC detection to specifically bind HER2 at distinct regions without interfering with its dimerization sites [5, 25]. Although our results did not directly confirm this, Nby-Aby theoretically enables dual-target detection of HER2 molecules, minimizing steric hindrance and potentially allowing for the detection of both HER2 monomers and dimers. This character may contribute to and explain the observed enhancement in HER2 scores and sensitivity compared to conventional methods. Additionally, integrating conventional IHC testing with Nby-Aby to comprehensively assess HER2 dimers and monomers could provide a more refined diagnostic approach for evaluating prognosis and recurrence risk in patients. For example, patients with HER2 scores of 1 + or 2 + in conventional IHC testing but 3 + in Nby-Aby testing might have different clinical outcomes compared to those with 3 + scores in both methods. However, this hypothesis requires further validation in subsequent studies.

The developed HER2 targeted therapy primarily functions in four ways: 1) Directly binding with HER2 and inhibiting its dimerization, such as pertuzumab [39]; 2) Binding with HER2 and activating ADCC effects, such as trastuzumab [40]; 3) Targeting the intracellular catalytic kinase domain of HER2, such as Lapatinib [41]; 4) Binding with HER2 followed by internalization of the ADC and release of cytotoxic payload to kill tumor cells, such as Ado-trastuzumab emtansine (T-DM1), and T-Dxd [32]. Considering the established efficacy and mechanisms of existing drugs, patients with Nby-Aby test results classified as 1 + or higher but zero in conventional IHC may be more suitable for treatment with T-Dxd, T-DM1, and Lapatinib. The reason is that these drugs may have the potential to eliminate tumor cells exhibiting HER2 dimerization and already activating downstream carcinogenic pathways, whereas conventional IHC methods may not currently detect HER2 dimerization states.

In conclusion, through binding different regions and overcoming steric hindrance of HER2 dimers in IHC, our research has introduced a novel IHC testing method using Nby-Aby to fully evaluate the levels of HER2 dimerization and resting. This method may serve as a valuable diagnostic tool for assessing HER2 expression more accurately and could provide a new strategy for predicting prognosis and guiding treatment in BC patients. However, this study has certain limitations. First, the sample size was relatively small, which may affect the generalizability of the results. Second, the study lacks long-term follow-up data to determine the clinical significance of increased HER2 scores detected using Nby-Aby than the traditional method. Further, the efficacy of guiding treatment for patients with positive and low expression results diagnosed by Nby-Aby in combination with traditional methods has not yet been validated through specific clinical drug interventions, and this is what we will continue to investigate further.

## Conclusions

Accurate HER2 assessment remains a challenge due to the presence of HER2 dimerization and mutations in IHC. This study demonstrates that the Nby-Aby fusion protein significantly enhances HER2 detection sensitivity in IHC by dual-targeting and binding distinct regions, without affecting its dimerization. Compared to the conventional IHC method, Nby-Aby improves HER2 IHC scores and enhances sensitivity. These findings suggest that through dual-targeting and binding different regions of HER2-ECD can reduce steric hindrance and improve diagnostic sensitivity in IHC. Future IHC detection of HER2 may require reagents take more focus on dual-targeting or targeting regions separate from the HER2 dimerization region to improve diagnostic accuracy.

## Supplementary Information

Supplementary Material 1: Figure S1. Schematic diagrams of plasmid constructs for the expression of HER2-targeting proteins. (A) The schematic diagram of pET21a(+)/HFn plasmid; (B) The schematic diagram of pET21a(+)/Aby-HFn plasmid; (C) The schematic diagram of pET21a(+)/Nby-Aby plasmid; (D) The schematic diagram of pET21a(+)/Nby-HFn plasmid.

Supplementary Material 2.

Supplementary Material 3.

## Data Availability

The datasets used in this study are available as follows: Publicly available data: The structural data used in this study, as described in the manuscript, can be accessed from the Protein Data Bank (PDB) under the following accession numbers: 1N8Y, 8HGO, 7MN5, 8U4K, 6OGE, 5MY6, and 3MZW. These datasets can be accessed at https://www.rcsb.org/. Restricted third-party data: The datasets obtained from Outdo Biotech Co., Ltd (Shanghai, China) are not publicly available due to ethical, legal, or licensing restrictions. However, they may be made available upon reasonable request and with appropriate permission from Outdo Biotech Co., Ltd. Data generated in this study: The datasets generated and analyzed during the current study are available from the corresponding author upon reasonable request.

## Abbreviations

HER: Human epidermal growth factor receptor
EGFR: Epidermal growth factor receptor (HER1)
BC: Breast cancer
IHC: Immunohistochemistry
ISH: In situ hybridization
ASCO: American Society of Clinical Oncology
CAP: College of American Pathologists
T-DXd: Trastuzumab deruxtecan
ECD: Extracellular domain
TMD: Transmembrane domain
ICD: Intracellular domain
Aby: Affibody
Nby: Nanobody
HFn: Human heavy chain ferritin
PLAs: Proximity Ligation Assays
FLIM-FRET: Fluorescence Lifetime Imaging Microscopy-Förster Resonance Energy Transfer
T-DM1: Ado-trastuzumab emtansine
