# Supplementary figures and images for: Dual-targeting and steric hindrance resolution in HER2 IHC: a novel approach to improve diagnostic sensitivity
Source: BMC Cancer. 2025 Jul 29;25:1231. doi: 10.1186/s12885-025-14553-7 (PMC12309212; doi:10.1186/s12885-025-14553-7)

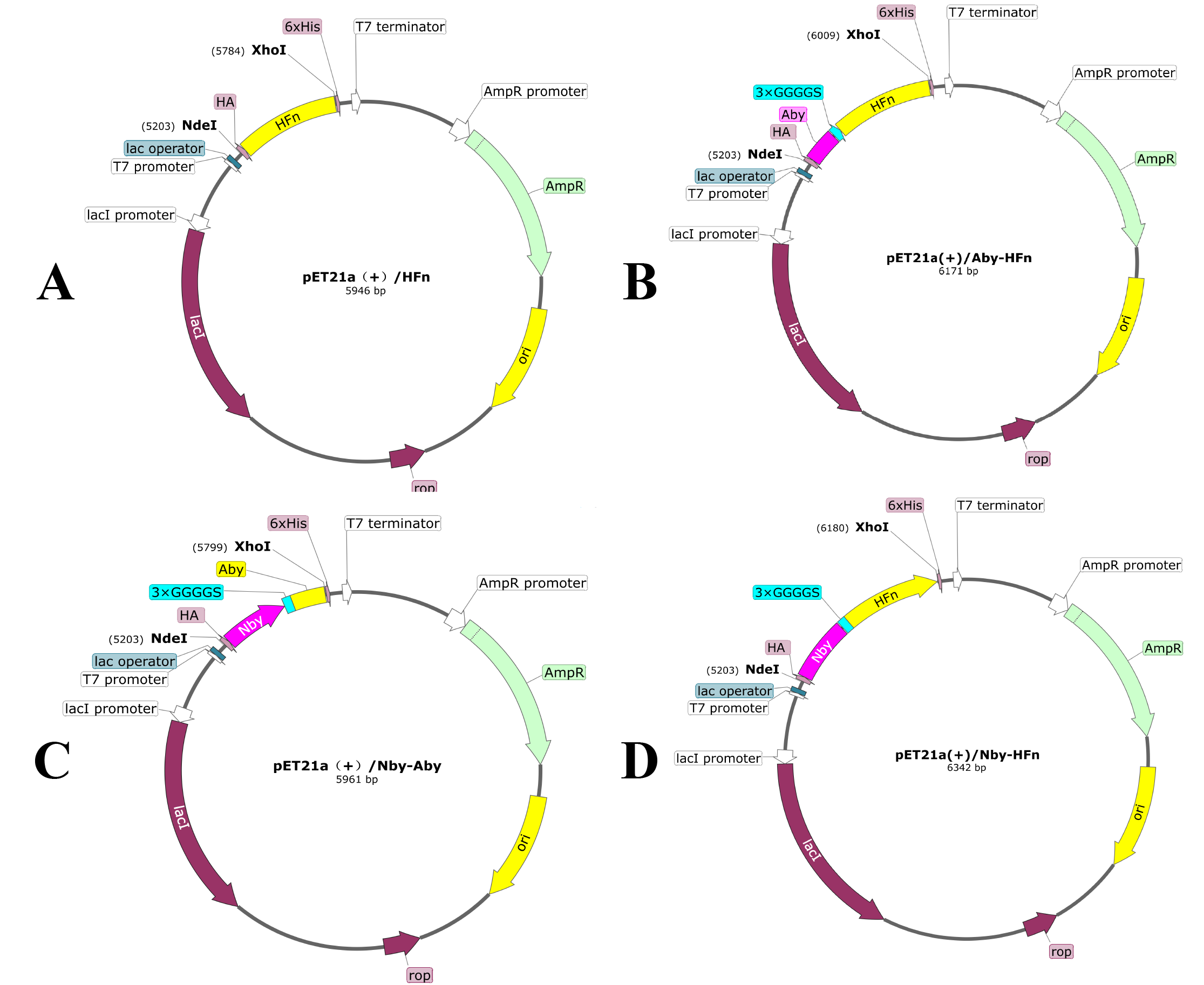

Supplement: Supplementary file 1 — Supplementary Material 1: Figure S1. Schematic diagrams of plasmid constructs for the expression of HER2-targeting proteins. (A) The schematic diagram of pET21a(+)/HFn plasmid; (B) The schematic diagram of pET21a(+)/Aby-HFn plasmid; (C) The schematic diagram of pET21a(+)/Nby-Aby plasmid; (D) The schematic diagram of pET21a(+)/Nby-HFn plasmid. [file 12885_2025_14553_MOESM1_ESM.tif]

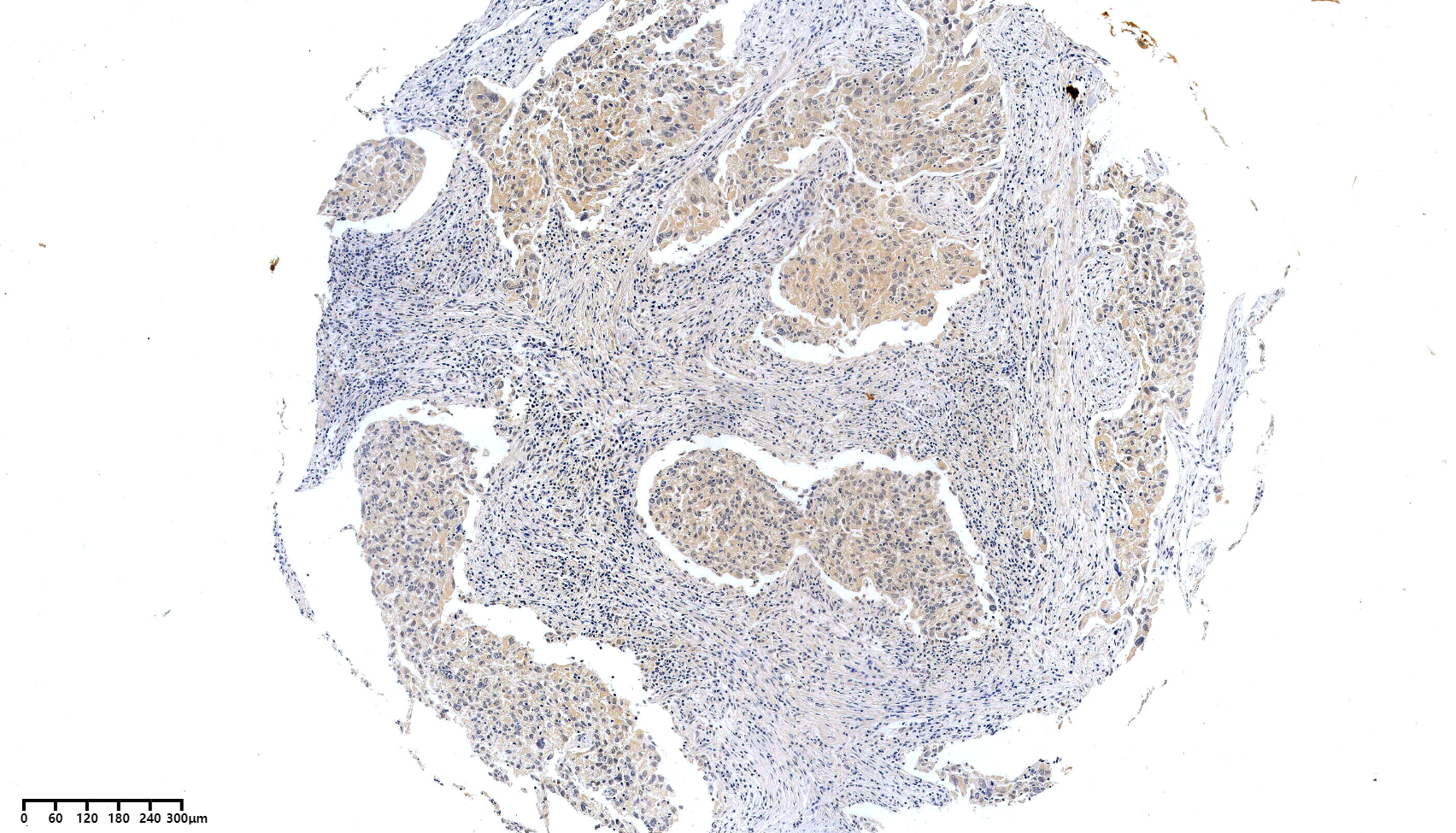

Supplement: Supplementary file 3 — Supplementary Material 3. [file 12885_2025_14553_MOESM3_ESM.zip › AA07++.tif]

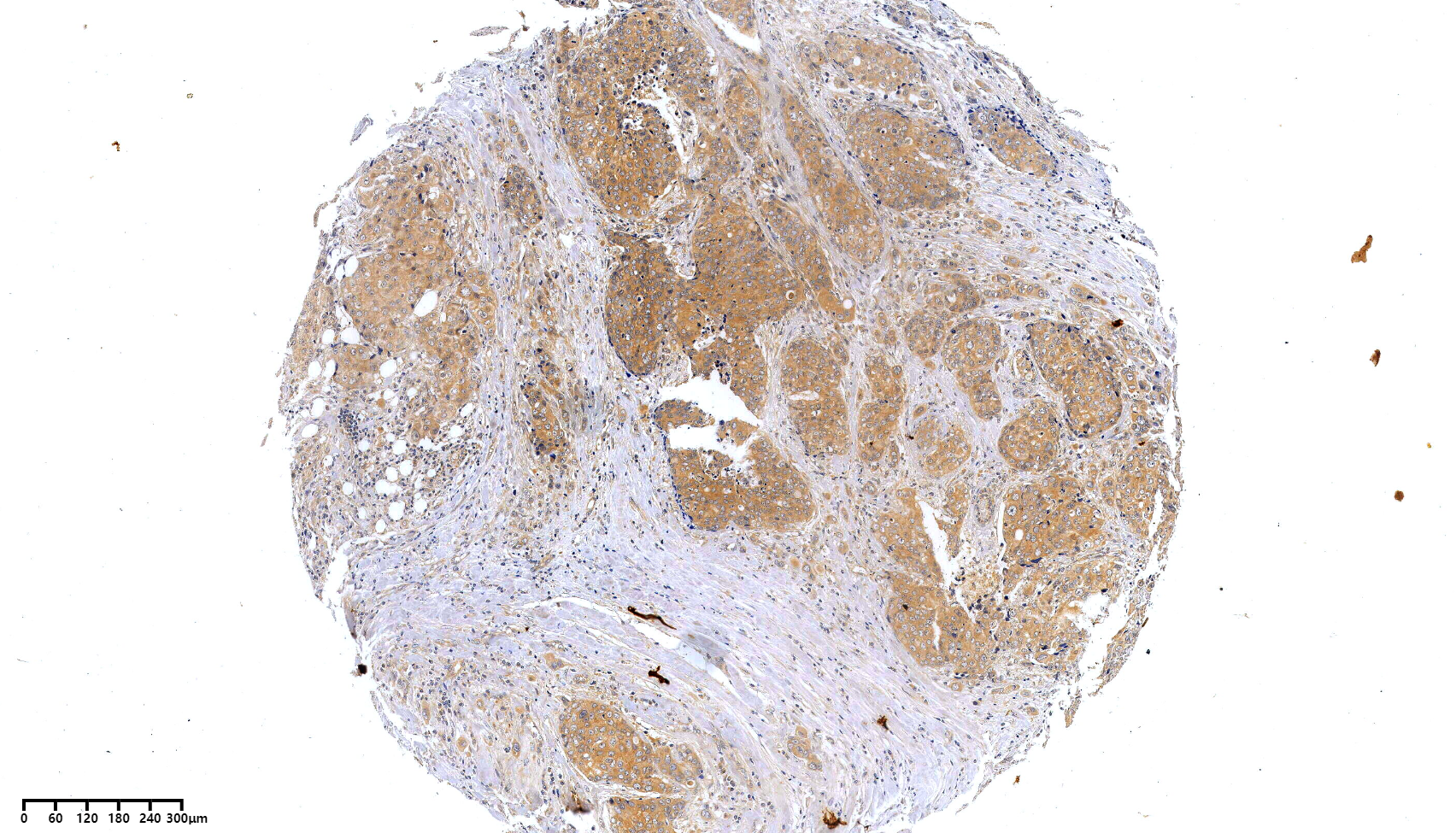

Supplement: Supplementary file 3 — Supplementary Material 3. [file 12885_2025_14553_MOESM3_ESM.zip › AB05+++.tif]

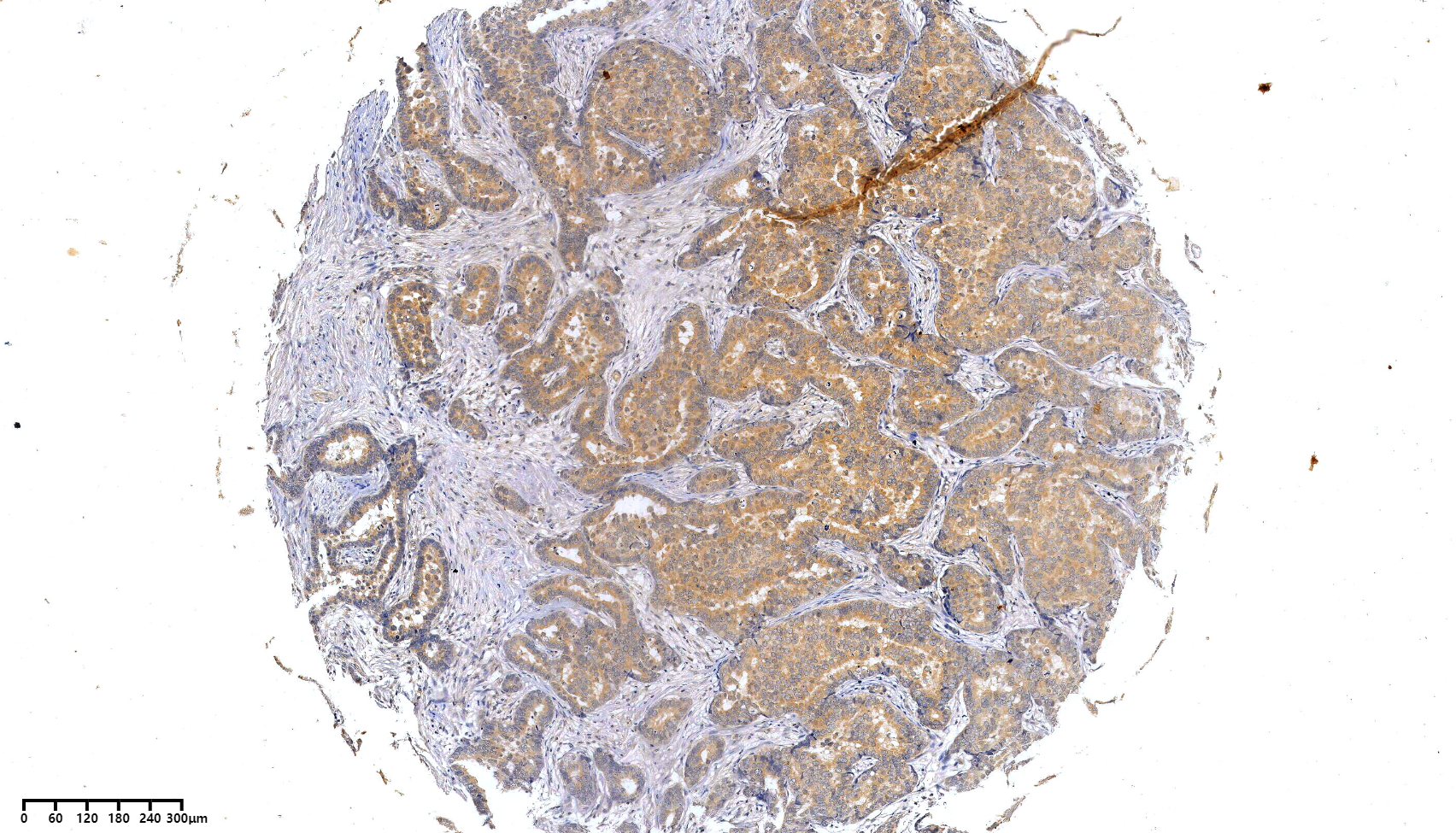

Supplement: Supplementary file 3 — Supplementary Material 3. [file 12885_2025_14553_MOESM3_ESM.zip › AD01+++.tif]

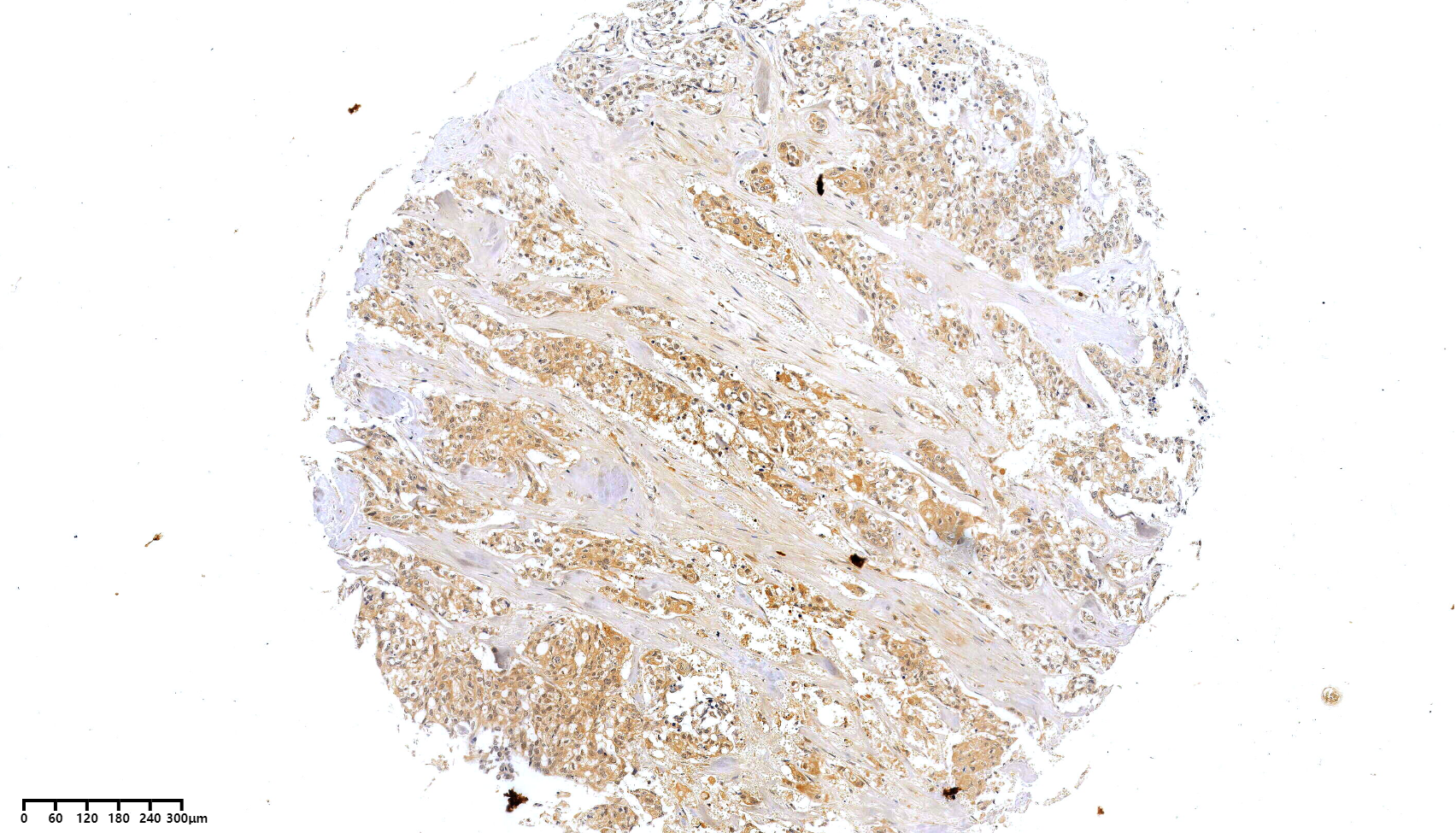

Supplement: Supplementary file 3 — Supplementary Material 3. [file 12885_2025_14553_MOESM3_ESM.zip › AG05++.tif]

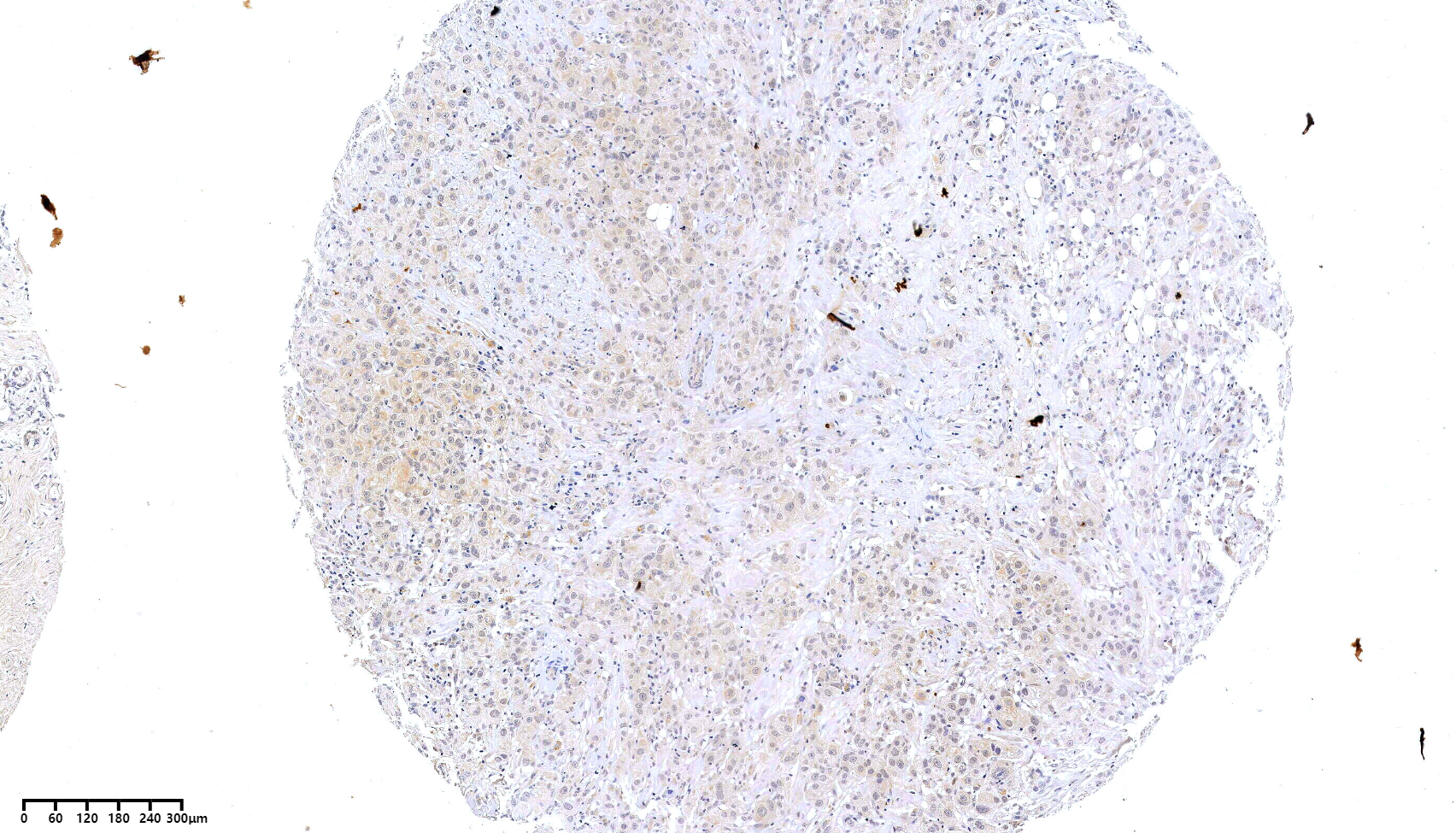

Supplement: Supplementary file 3 — Supplementary Material 3. [file 12885_2025_14553_MOESM3_ESM.zip › BC13++.tif]

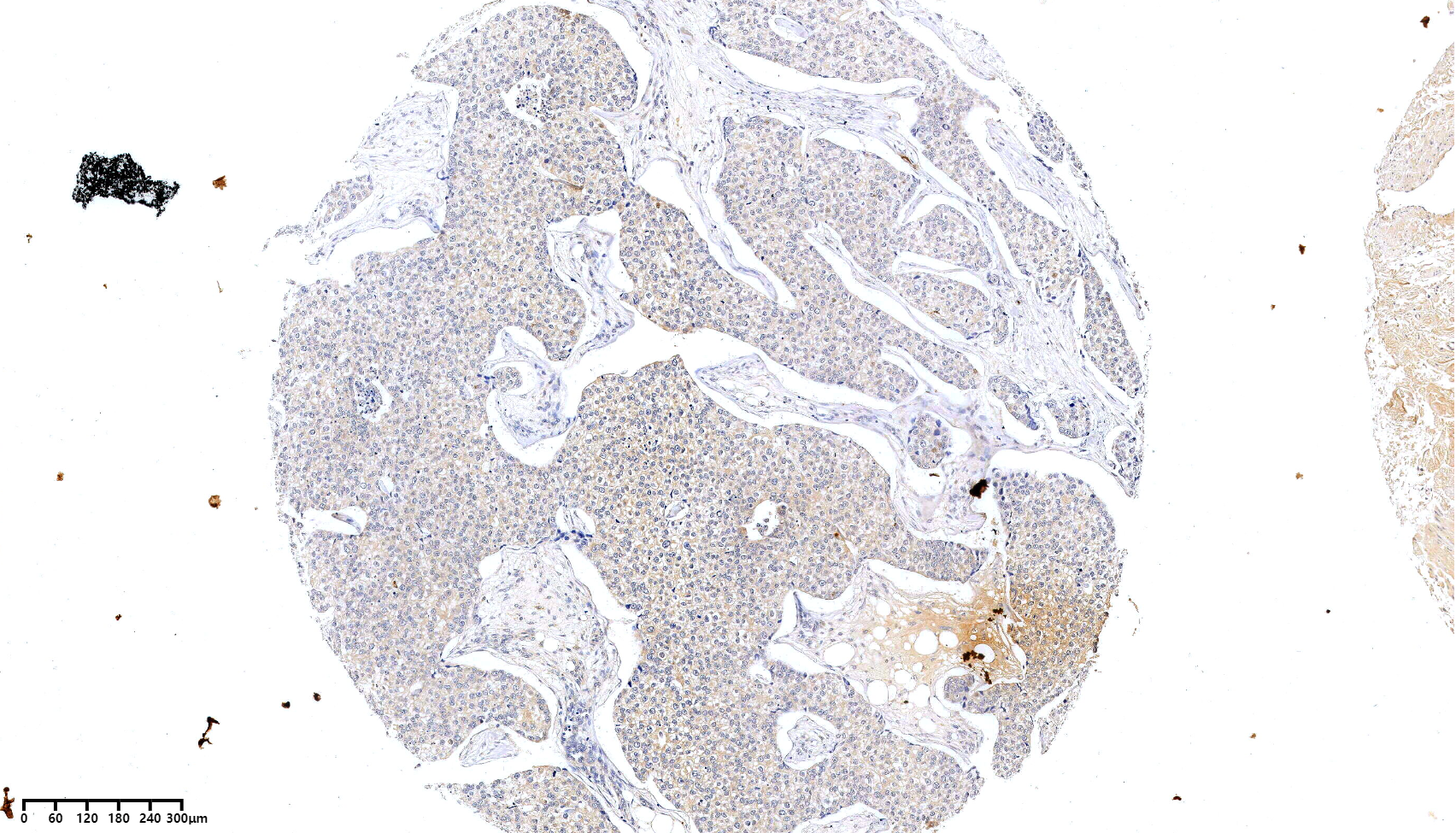

Supplement: Supplementary file 3 — Supplementary Material 3. [file 12885_2025_14553_MOESM3_ESM.zip › BF15+++.tif]

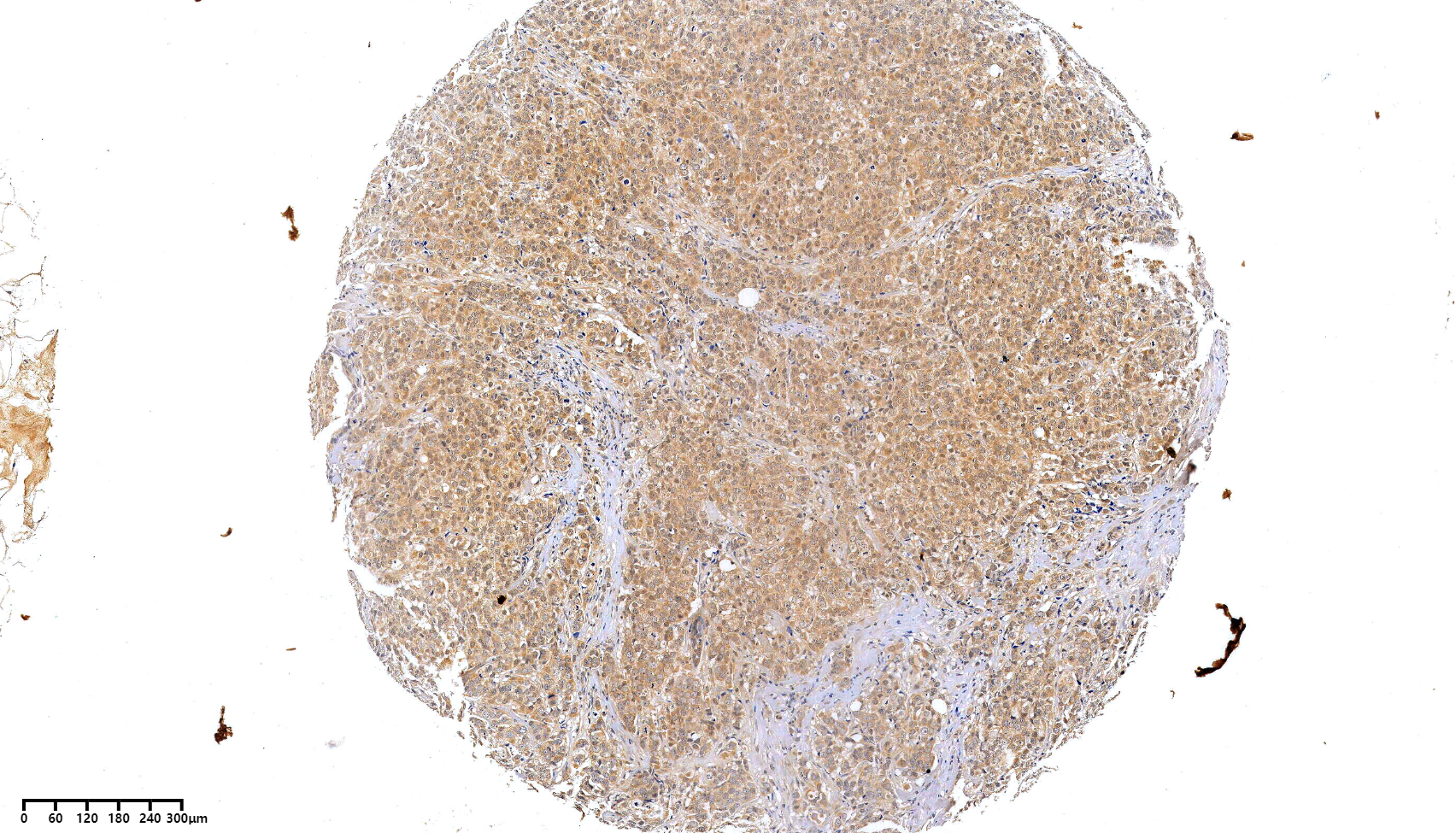

Supplement: Supplementary file 3 — Supplementary Material 3. [file 12885_2025_14553_MOESM3_ESM.zip › BG05+++.tif]

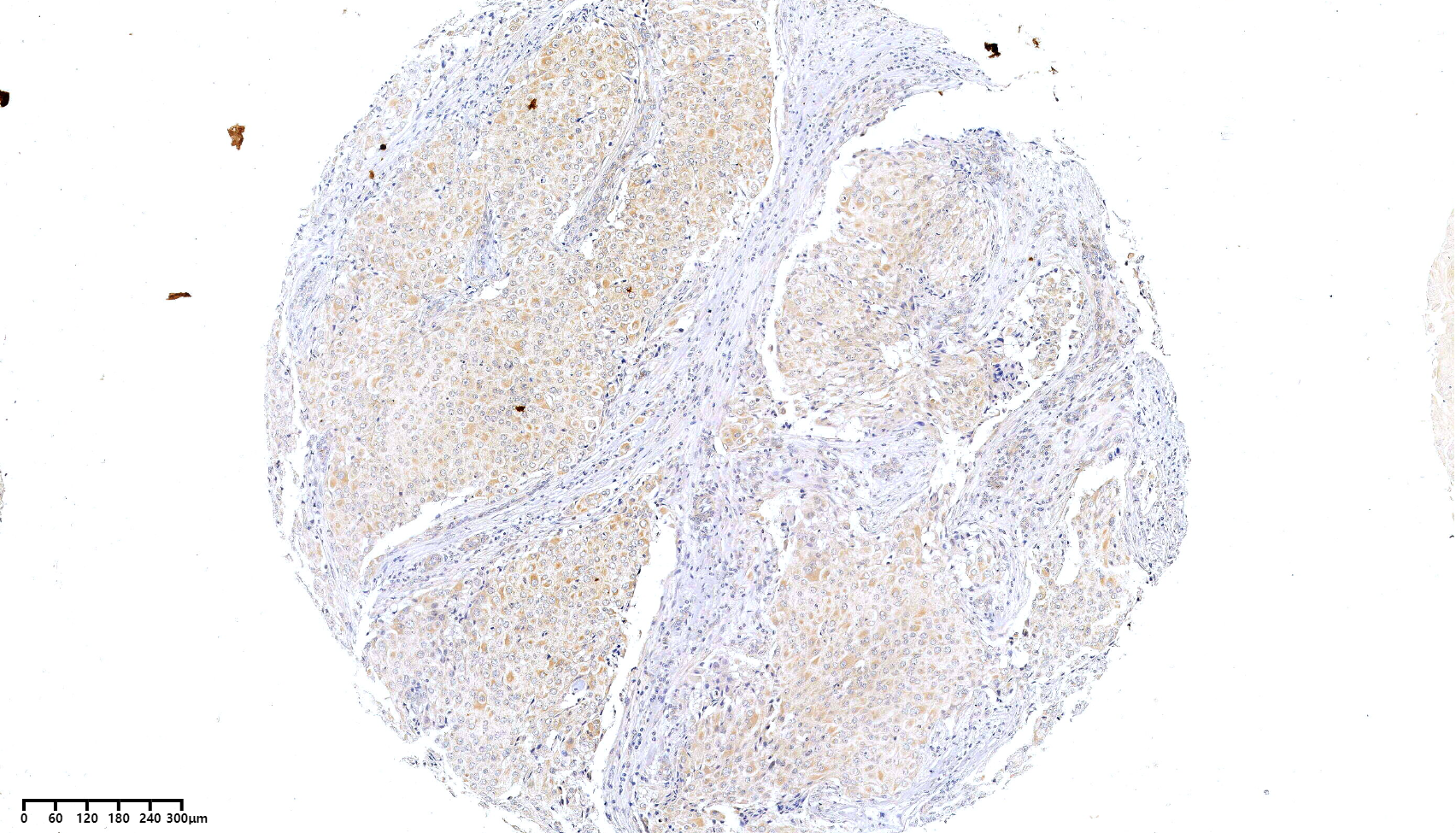

Supplement: Supplementary file 3 — Supplementary Material 3. [file 12885_2025_14553_MOESM3_ESM.zip › BG15+++.tif]
